# The dual structure of ‘total pain’: multidimensionality and holism in the foundational concept of palliative care

**DOI:** 10.3389/fsoc.2026.1985788

**Published:** 2026-10-16

**Authors:** David Fajardo-Chica

**Affiliations:** 1 Institute for Philosophical Research, National Autonomous University of Mexico, Mexico City, Mexico; 2 Palliative Care Working Group, Global Studies Seminar, Faculty of Medicine, National Autonomous University of Mexico, Mexico City, Mexico

**Keywords:** Cicely Saunders, holism, multidimensionality, palliative care, philosophy of suffering, suffering, total pain

## Abstract

The concept of ‘total pain’, introduced by Cicely Saunders, is often treated as a historical milestone of the hospice movement rather than as a live theoretical resource. I argue instead that it is a concept with a definite architecture that remains operative as an implicit theory of suffering in contemporary palliative care. This architecture takes the form of two theses: an etiological thesis of multidimensionality, on which suffering arises from impairments across multiple dimensions of the person (physical, psychological, social, spiritual, among others), and an experiential thesis of holism, on which suffering is lived as affecting the person as a whole rather than a sum of discrete problems. I show that this dual structure is legible across the WHO definition of palliative care, the field’s consensus definition, its validated measurement instruments, and the Lancet Commission’s concept of serious health-related suffering. Each thesis plays a distinct role: multidimensionality supports a multidisciplinary approach to suffering, while holism supports respect for patient autonomy by giving due weight to the patient’s perspective. I then ask whether two alternative conceptions—the objective, flourishing-based view and the thin phenomenological view—could perform the same work, and argue that the former secures clinical tractability at the cost of autonomy, while the latter centers the patient’s experience without securing its link to symptoms. Finally, I address the objection that the dual structure cannot explain how distributed impairments become a unified experience, drawing on recent models in the philosophy of mind to show that it can. ‘Total pain’, then, is a conceptual compromise enabling care that is at once clinically effective and ethically person-centered.

## 1 Introduction

‘Total pain’ is arguably the founding concept of palliative care. Saunders (1964, 2001) introduced it to describe the experience of patients at the end of life, whose distress could not be captured by physical symptomatology alone (Krawczyk and Richards, 2018). The concept was decisive in the development of the hospice model, which later evolved into the palliative care model (Clark, 2000). Yet ‘total pain’ tends to be treated in one of two deflationary ways: as a clinical metaphor—a vivid reminder that dying patients hurt in more ways than one, carrying no theory of its own—or as a historical milestone, an episode in the disciplinary formation of a medical field. On the first reading the concept has no conceptual content to reconstruct; on the second, whatever content it had belongs to the prehistory of the field rather than to its present. Recent reconsiderations of the concept in this journal have questioned the extent to which its original commitments persist in contemporary practice (Rattner, 2023; Timm, 2023; Krawczyk et al., 2025; Tarditi, 2026), raising the question of how ‘total pain’ should be understood today.

In this article I argue that ‘total pain’ is a concept with an architecture that continues to operate as the field’s implicit theory of suffering in contemporary palliative care. It surfaces across clinical guidelines, validated measurement instruments, and normative discourses (World Health Organization, 2002; Radbruch et al., 2020). It functions as a shared framework that enables coordination, evaluation, and communication because it encodes a specific account of what suffering is and where it comes from, one this article sets out to reconstruct. This reconstruction also speaks to the recent critique that ‘suffering’ is too ambiguous to be theoretically useful (Nelson et al., 2025). Although the concept of suffering remains indeterminate in general, I defend that the conception expressed in ‘total pain’ has a determinate structure.

The architecture of ‘total pain’, I argue, consists of two theses that operate at different levels. The first is etiological: suffering arises from impairments across multiple dimensions of the person, including the physical, psychological, social, and spiritual. I call this the thesis of multidimensionality. The second is experiential: suffering is lived not as a collection of isolated problems but as something that affects the person as a whole. I call this the thesis of holism. ‘Total pain’ is the conjunction of both. Multidimensionality makes suffering tractable for clinical intervention, while holism keeps care anchored in the lived experience of the person.

Elucidating this dual structure is a valuable theoretical objective because ‘total pain’ does not fit existing conceptions of suffering in the philosophical and bioethical literature. Following Kious (2022), we can distinguish three families of theory: *objective* theories, on which suffering is the absence of objective flourishing (van Hooft, 1998); *feeling-based* theories, which equate suffering with aversive sensation (Mayerfeld, 1999; Edwards, 2003); and *value-based* theories, on which suffering is distress at threats to what one cares about, as in Cassell’s influential account (Cassell, 1982, 1991a). ‘Total pain’ sits closest to the value-based family, but it is not exhausted by it, since it also commits to a specific multidimensional etiology that value-based accounts leave open. I therefore focus on the two conceptions from which ‘total pain’ genuinely diverges: the objective and the feeling-based phenomenological conception of suffering. By making this structure explicit, we can better understand the conceptual foundations of palliative care and clarify how ‘total pain’ relates to other theoretical models of suffering.

I develop the argument in six sections. In Section 2, I reconstruct the dual structure of ‘total pain’ from Saunders’s work and show how Eric Cassell’s influential theory of suffering systematizes its holistic thesis. In Section 3, I show that this dual structure remains operative in the contemporary field: the WHO definition of palliative care, as well as the consensus definition, its measurement practices, and the global health concept of serious health-related suffering. In Section 4, I examine the distinct normative roles that each component plays: multidimensionality supports the multidisciplinary model of intervention, while holism supports respect for autonomy. In Section 5, I compare ‘total pain’ with the objective and feeling-based theories of suffering, arguing that neither can perform both functions. In Section 6, I address the objection that the dual structure of ‘total pain’ is incoherent, drawing on recent theories in philosophy of mind that explain how suffering can result from distributed impairments. In Section 7, I offer a final discussion and draw some conclusions.

## 2 The dual structure of ‘total pain’

Saunders (1964, 2001) introduced ‘total pain’ to make sense of the recurrent clinical observation that patients at the end of life suffer not only from physical pain, but also from psychological, social, and spiritual distress, and this distress affects them as whole persons. The concept has two faces. On one face, it recognizes multiplicity: the pain of the patient has physical, psychological, social, and spiritual components, each of which can be identified and addressed. On the other face, it unifies: what the patient undergoes is one experience, a totality, not a bundle of parallel complaints. Patients themselves report this unity. They say that everything is affected, or that they no longer feel like themselves (Saunders, 1964). These expressions do not describe each impairment separately; they convey the sense of being overwhelmed as a whole.

These two faces correspond to two theses that jointly constitute the concept:

*Multidimensionality (etiological thesis):* Suffering arises from impairments across multiple dimensions of the person—paradigmatically the physical, psychological, social, and spiritual.

*Holism (experiential thesis):* Suffering is experienced as affecting the person as a whole, rather than as a collection of isolated problems.

Throughout, I take these four dimensions—physical, psychological, social, and spiritual—as the canonical core of the concept, following Saunders’s (1964, 2001) own formulations. But the thesis of multidimensionality is not committed to a fixed inventory. The term *dimension* remains intentionally broad. It refers to structured aspects of personhood—bodily, interpersonal, cultural, historical, moral, and, as Saunders herself often added, spiritual—that can be meaningfully affected by illness or its consequences. The thesis holds that suffering originates in impairments to one or more of these dimensions of the person. This openness reflects the clinical intuition, already present in Saunders, that suffering exceeds the physical and includes the broader human context.

The most influential systematization of the holistic thesis is Eric Cassell’s theory of suffering as a “threat to the person” (Cassell, 1982, 1991a). Cassell’s account has been highly influential in the health sciences, though not uncontested (den Hartogh, 2017; Duffee, 2020). According to his theory, suffering occurs when a person perceives a threat to their sense of personhood; it is the person, not just the body, who suffers. Cassell identifies a wide range of dimensions of the person that can be threatened (Cassell, 1982).^1^ Any harm in these dimensions can contribute to a general sense of threat, which constitutes suffering. Cassell’s theory articulates, with a philosophical vocabulary of personhood, the same dual structure that Saunders had already built into ‘total pain’. There is, however, a difference in what role the dimensions play in the two accounts. In Cassell, they are aspects of the person that can be threatened; in ‘total pain’, they are also causal fronts of clinical intervention, each answerable to a distinct form of care. Despite the frequent identification of Cassell’s view as the dominant theory of suffering in the field (den Hartogh, 2017; Duffee, 2020), its underlying architecture is Saundersian.

A further elaboration of the same structure is the integrated view of suffering proposed by Krikorian and Limonero (2012), who emphasize how problems in one dimension can intensify difficulties in others, leading to a general increase in suffering. Their account adds a dynamic of interaction in which impairments amplify one another and overwhelm the person’s capacity to adapt. Again the two theses appear together—dimensional etiology, integrated experience—but now with an explicit account of how the dimensions combine, a point I return to in Section 6.

Two clarifications prevent misreadings of the dual structure, and together they show why neither thesis can do the concept’s work alone. Each clarification guards one thesis against a characteristic distortion. The first clarification protects holism against an additive misreading. ‘Total pain’ does not claim that suffering requires impairments in multiple personal dimensions. A serious impairment in a single domain (e.g., severe pain, profound grief, or social isolation) may suffice. The relevant criterion is whether the person experiences the disturbance as affecting them as whole. Multidimensionality describes a common etiological pattern, while suffering derives its unity from its impact on the person and may therefore arise from a disturbance in a single dimension. The second clarification protects multidimensionality against a trivializing misreading. ‘Total pain’ does not hold that any set of minor impairments across multiple domains amounts to suffering. A stiff joint, thinning hair, or reduced stamina may involve several dimensions, but unless they are perceived as affecting the person as a whole, they do not constitute suffering. The etiological thesis does not assert that any dimensional damage feeds suffering, but that suffering, when it occurs, originates in impairments that the person experiences as bearing on them as a whole. Holism supplies the condition that the etiology alone cannot.

Suffering tends to reverberate across domains. A major impairment, such as a diagnosis of terminal illness, can undermine social bonds, emotional stability, and even bodily function (Krikorian and Limonero, 2012). From the holistic perspective, the personal dimensions are interdependent structures. A rupture in one domain may destabilize the person as a whole, thereby affecting others indirectly. This reinforces the claim, central to ‘total pain’, that suffering is not merely additive but integrative in its structure. Cassell (1982, p. 640) noted that patients do not distinguish the sources of their suffering as clinicians do; suffering appears to them not as a sum of individual impairments, but as a disruption of the person’s sense of integrity and coherence. ‘Total pain’ leaves the principle of integration unspecified, raising the question of how an impairment in one dimension comes to affect the whole person. Some answers are available (e.g., through a threat to the person’s integrity, a disruption to mental life, or an alienation from one’s world), and I consider them in Section 6.

## 3 ‘Total pain’ as the operative framework of contemporary palliative care

The dual structure of ‘total pain’ concept reconstructed in Section 2 surfaces wherever the contemporary field defines, measures, or globally assesses suffering. Consider first the field’s definitions. The World Health Organization defines palliative care as an approach that improves quality of life through the prevention and relief of suffering, by means of the identification, assessment, and treatment of pain and other problems—physical, psychosocial, and spiritual (World Health Organization, 2002). The enumeration of problem-domains expresses the multidimensional thesis. However, multidimensional vocabulary is not the exclusive legacy of the ‘total pain’ concept. It converges with other challenges to biomedical reductionism, notably Engel’s biopsychosocial model (Engel, 1977), a model of disease that frames its causes hierarchically, as causally integrated levels —biological, psychological, and social— but without the holistic thesis that the resulting impairments are undergone as a single experience of the person.

The WHO’s characterization of palliative care also reflects the holistic thesis. WHO Europe describes palliative care as addressing patients’ “holistic needs—physical, psychosocial, and spiritual” (World Health Organization Regional Office for Europe, n.d.), and WHO EMRO as care directed at “the whole person” rather than at isolated symptoms (World Health Organization Regional Office for the Eastern Mediterranean, 2025). The more recent consensus-based definition of the field (Radbruch et al., 2020) makes this explicit at the level of the definition itself, characterizing palliative care as the “active holistic care” of persons with serious health-related suffering across physical, psychological, social, and spiritual dimensions. This holistic vocabulary, too, is not the exclusive legacy of the ‘total pain’ concept. It resonates with the longstanding whole-person tradition in nursing (Nightingale, 1860), which advocates a holistic approach to the patient as a model of care. Yet this is a stance toward how the patient should be attended to, not an account of the nature of suffering.

The measurement practices of the field exhibit a consistent pattern. Internationally validated instruments for assessing suffering at the end of life have been systematically reviewed (Gutiérrez-Sánchez et al., 2020), and most are built on a multidimensional design that decomposes suffering into discrete symptoms (Fajardo-Chica, 2023). Such instruments typically assess a range of physical impairments—pain, dyspnea, constipation—alongside psychological distress, social and personal difficulties, and spiritual suffering (MacAdam and Smith, 1987). At the same time, other instruments are designed to capture the global, integrated character of the experience. The SOS-V (Ruijs et al., 2009) explicitly distinguishes the intensity of individual symptoms from the overall unbearability of suffering, which need not track any single symptom. The PRISM goes further in this holistic direction (Büchi and Sensky, 1999), it is a nonverbal instrument on which the patient represents the distance between the self and the illness, it measures suffering not as a profile of impairments but as the global encroachment of illness upon the person. What matters for my argument is that the two kinds of instrument measure suffering in two registers, one decomposing it into discrete domains and the other assessing it as a global burden on the person. That the field has found both registers indispensable is consistent with the dual structure of the ‘total pain’ concept reconstructed in Section 2.

In global health, the assessment of suffering reflects the same structure. The Lancet Commission on Palliative Care and Pain Relief introduced the concept of serious health-related suffering (SHS) to establish a proxy for palliative need and to measure the state of palliative care across regions of the world. SHS is defined as suffering associated with illness or injury of any kind that becomes serious when it cannot be relieved without professional intervention and when it compromises physical, social, spiritual, and/or emotional functioning (Knaul et al., 2018). This concept has since been incorporated into the consensus definition of the field (Radbruch et al., 2020). The methodology of the Lancet Commission, later adopted by the Global Atlas of Palliative Care (Connor, 2020), identified common diseases and their usual symptoms, producing a list of eleven physical and four psychological symptoms.^2^ The assessment of SHS proceeds through these fifteen symptoms, while the very notion of suffering that these symptoms are meant to capture retains the holistic orientation of ‘total pain’, in which it is the person whose functioning is compromised. Definitions, instruments, and global metrics thus converge on the same dual structure. Its presence remains legible across the contemporary field, which also continues to generate theoretical and empirical work, as the concept is extended to new populations and contexts, such as dementia (Field-Richards et al., 2025), cancer survivorship (Krawczyk et al., 2025), social suffering among marginalized groups (Graven et al., 2023; Timm, 2023), and even the indirect victims of enforced disappearance (Tarditi, 2026).

## 4 The normative roles of the dual structure

Each of the two theses of ‘total pain’ grounds a distinct normative commitment. Multidimensionality grounds the clinical model of intervention; holism grounds an ethical demand centered on the person as a whole. The former secures clinical tractability; the latter secures respect for autonomy. These roles are not interchangeable, and neither reduces to the other: they let care move between a clinical stance, which identifies and addresses discrete causes, and an ethical one, which attends to the overall shape of the person’s experience. ‘Total pain’ makes room for both, without subordinating either. The following sections take each in turn: the clinical role of multidimensionality (4.1) and the ethical role of holism (4.2).

### 4.1 The clinical role of multidimensionality

The multidimensional thesis supports a diagnostic and pragmatic stance toward suffering. It allows clinicians to map the various domains in which impairments arise—physical, psychological, social, spiritual—and to address each through appropriate expertise. Crucially, these domains are qualitatively heterogeneous. Relieving physical pain, addressing social isolation, and treating psychological distress require different forms of expertise, including pharmacology, social intervention, and psychotherapy. Because the sources of suffering belong to different orders, no single expertise can reach them all. This is what grounds the multidisciplinary model of palliative care, which the field’s own definitions translate into a “team approach” (World Health Organization, 2002; Radbruch et al., 2020) deploying not only physicians and nurses but also psychologists, social workers, and spiritual care providers (Fajardo-Chica, 2020).

The division of labor is the standard model of palliative care, in which each discipline attends to a distinct kind of need while working collaboratively with the others (Teoli et al., 2023). Their roles are differentiated but overlapping (Saks et al., 2026). Each dimension may call not only for different forms of expertise but also for a different approach. For example, care for the spiritual dimension, but also for the psychological and social ones, calls for a cultural sensitivity (Amalia et al., 2025) which is not generally part of what is expected when addressing physical impairments.

This model breaks with the limitations of the traditional biomedical paradigm, which tends to treat suffering as a proxy for physical symptom severity—a reduction Cassell traced to the persistence of mind–body dualism in medicine (Cassell, 1982). The multidimensional thesis of ‘total pain’ validates forms of distress that have no obvious somatic correlate—feelings of abandonment, loss of meaning, isolation—thereby expanding the range of what counts as clinically relevant. Historically, this was precisely the innovation of Saunders’s concept: it authorized the clinic to treat as pain—and therefore as its proper object—what the biomedical model had classified as extraneous to it. As Clark (1999) notes, this authorization also expanded clinical attention to the whole person, extending disciplinary authority into areas that had previously fallen outside its scope.

### 4.2 The ethical role of holism

The holistic thesis supports an ethical stance toward suffering by emphasizing that suffering is undergone as a whole. According to the holistic thesis, impairments are lived as affecting the person as a whole. This mode of experience demands a kind of attention that goes beyond treatment and involves recognition. Holism frames the patient not as a bearer of symptoms, but as a person whose capacity for coherence is at stake. This is the aspect of ‘total pain’ that Cassell’s theory made explicit: it is persons, not bodies, who suffer (Cassell, 1982). It helps explain why patients may experience their suffering as affecting their whole life, even when only a few domains are visibly affected. As Saunders’s patient put it: “The pain began in my back, but now it seems that all of me is wrong” (Saunders, 1964).

This holistic character links the ‘total pain’ conception of suffering to respect for autonomy, but we should distinguish three distinct claims about how the two are related. First, the sufferer has privileged epistemic authority over what their suffering is. Second, the sufferer’s values and perspective carry particular weight in determining what would count as relief. Third, the sufferer has decisional authority over what should be done, which is not supported by the holism thesis of the ‘total pain’ concept.^3^ The first claim follows from the first-personal character of suffering. Because suffering is lived as affecting the person as a whole, its full significance is accessible only from the perspective of the person who experiences it. A clinician can observe a symptom from the outside, but the significance of its effects on the person’s life cannot be independently established from outside. On Cassell’s account, indeed, the sufferer has a kind of final authority in establishing what they suffer, even though others may identify the threats they face and judge how they themselves would suffer under similar circumstances (Cassell, 1991b).

The second claim concerns the role of the patient’s values in determining what counts as relief from their suffering. Discussions of autonomy have emphasized the importance of patients’ values and preferences in decisions about their care, including in palliative settings (Woods, 2005; Beauchamp and Childress, 2009; Houska and Loučka, 2019; Rechenmacher, 2025). The holistic thesis connects the experience of suffering to respect for autonomy. If suffering involves how a person experiences and makes sense of their life, then the patient’s narratives, values, and life projects become relevant to determining what would constitute an adequate response to it. This consideration is relevant to decisions about care planning, informed consent, withdrawal of life-sustaining treatment, and palliative sedation, among other aspects of end-of-life care.

‘Total pain’ implies that patients have epistemic privilege regarding their suffering, but this does not by itself establish decisional authority. Respect for autonomy ordinarily requires that the patient’s preferences guide care, but professional judgement may legitimately depart from them when decision-making capacity is impaired, preferences are unstable or ambivalent, or other ethically relevant considerations apply, as in situations common in palliative care settings, such as delirium and acute psychological distress. Holism therefore does not grant the patient a veto; instead, it requires professionals to justify departures from the patient’s perspective. The distinction between care and paternalism does not turn simply on whether a professional follows the patient’s will. As Rechenmacher (2025) argues, it depends on several factors, including the patient’s actual will, the professional’s intention, the means employed—persuasion, nudging, incentives, coercion, among others—and the justification offered for departing from that will.

The ethical significance of holism does not require abandoning a clinically useful focus on individual symptoms. ‘Total pain’ preserves the insight that such symptoms acquire their full significance within the patient’s experience as a whole. Thus, identifying and treating discrete impairments need not conflict with understanding suffering as a whole-person experience. The holism in ‘total pain’ also carries an ambivalence because opening new personal domains to clinical intervention can create new forms of vulnerability, including those associated with expectations of patient self-disclosure (cf. Clark, 1999). This ambivalence introduces a tension into the ethical significance of holism, but its further implications lie beyond the scope of the present discussion.

## 5 ‘Total pain’ and rival conceptions of suffering

Suppose one accepts that ‘total pain’ has the dual structure described so far. It could still be objected that some other conception of suffering would serve palliative care better. This objection adds to existing criticisms of aspects of this conception of suffering, ranging from concerns about incorporating the spiritual dimension into clinical practice (Randall and Downie, 2006) to criticisms of Cassell’s account (Duffee, 2020). If we look to recent taxonomies of suffering for such an alternative, two of the three families distinguished by Kious (2022) stand out as candidates: an objective, flourishing-based view (van Hooft, 1998, 2000) and a phenomenological view, encompassing both feeling-based and intuitive accounts (Edwards, 2003; Kious, 2022). I will now examine whether either can perform the conceptual work that ‘total pain’ performs in palliative care.

Let us first recall what that work is. ‘Total pain’ links two elements in the relationship between the palliative care model and suffering: the clinical identification of causal chains for intervention (the multidimensional thesis) and the ethical consideration of the patient’s personal experience and autonomy (the holistic thesis). Alleviating suffering involves intervening in these causal chains; attending to suffering requires respecting the patient’s experience and their authority over it. The question is whether the alternative conceptions offer the same dual benefit.

### 5.1 The objective conception

Van Hooft’s (1998, 2000) objective conception of suffering rests on an account of the nature of the beings who suffer. On this view, living beings have various levels of existence that can be disrupted, preventing their proper development. Drawing on an Aristotelian account of the soul, van Hooft’s framework identifies four levels of human functioning: the biological (basic bodily needs), the appetitive (desires such as hunger or sexual drive), the deliberative (rationality and personal projects), and the contemplative (the meaning given to life). Each level tends toward a goal or telos, and suffering is defined as the frustration of the tendency toward fulfillment of these aspects of our being (van Hooft, 1998, p. 126). A person may suffer from conditions such as addiction, poor nutrition, or social deprivation even if they do not perceive these as threats to themselves—and, in van Hooft’s strongest formulation, may suffer without any awareness of it at all.

The objective conception aligns with part of what the multidimensional thesis of ‘total pain’ does. It allows for the identification of a wide range of causal pathways to suffering, including those the individual does not directly perceive. In this respect van Hooft’s view captures the medical intuition that relieving symptoms relieves suffering, since those symptoms frustrate the flourishing of the person along some dimension. But the resemblance is partial. Van Hooft’s dimensions are levels of objective functioning whose frustration constitutes suffering independent of any state of consciousness of the person; the multidimensional thesis of ‘total pain’, by contrast, treats dimensions as causal sources whose significance is realized in the patient’s experience. The objective view recognizes multiple causes of suffering but detaches them from the lived experience.

This detachment is precisely why the objective conception is ill suited to patient autonomy. Because suffering is fixed independently of the person’s own experience, principles, or values, it can be identified—and acted upon—without reference to the patient’s point of view (Edwards, 2003, p. 62). Van Hooft embraces this consequence, on his account, medicine relieves suffering by curing malady and relieving pain, and there is “no medical mission to confront the spiritual condition of the patient” (van Hooft, 1998, p. 125). Whatever its merits as a theory of suffering in general, this is the opposite of what the holistic thesis secures. The objective view can reproduce the paternalism that palliative care seeks to avoid, licensing providers to judge a patient’s suffering, and the decisions it warrants, better than the patient. The objective conception, in short, can approximate the multidimensional thesis of ‘total pain’, but not its holistic one.

### 5.2 The phenomenological conception

The phenomenological conception of suffering focuses on the qualitative side of the experience—how it feels to suffer. I use “phenomenological” as an umbrella for a family of experience-centered accounts that differ in how much they build into that experience. The thin conception stays close to the felt quality of suffering. It encompasses the feeling-based view, on which to suffer is to undergo unpleasant, distressing states (Mayerfeld, 1999), and Edwards’s intuitive account, which, based on an analysis of the ordinary use of the word “suffering,” identifies it by its felt unpleasant character, its duration, and its dominance of a person’s mental life (Edwards, 2003). A richer strand goes further: embodied and lifeworld phenomenologies, on which suffering is a disturbance in one’s relation to body, lived world, and values (Svenaeus, 2014). The objection I raise tells against the thin conception, not against the richer one, which—as I discuss in Section 6—the ‘total pain’ conception of suffering can draw on rather than reject.

The thin phenomenological conception shares with ‘total pain’ the view that suffering is a first-person experience in which the patient’s perspective is central. This is the very feature the holistic thesis exploits in supporting autonomy. But the thin phenomenological view has two limitations. The first is epistemic. Because suffering is identified with how it feels, Edwards (2003, p. 60) observes that we may judge objectively whether a person is undergoing a physical ordeal, yet—since such ordeals carry different significance for different people—we cannot thereby judge whether they are suffering. However, not every proponent concedes this, Mayerfeld (1999) holds that feeling-based suffering is an objective psychological condition open to measurement. But even granting measurability, the deeper problem remains.

That deeper problem constitutes the second limitation. The phenomenological conception does not guarantee any relationship between suffering and symptoms. If suffering is primarily a feeling, nothing in the concept explains why particular impairments should give rise to it. In contrast, the multidimensional thesis of ‘total pain’ establishes causal links between symptoms and suffering, which is what makes suffering tractable for intervention. The thin phenomenological conception attends to the patient’s experience, but holism does more than register that suffering feels bad, it holds that suffering is lived as affecting the person as a whole. ‘Total pain’ couples this experience with a multidimensional etiology, promoting a focus on the patient’s experience without abandoning the scientific understanding of suffering that the subjective approach forgoes (Edwards, 2003, p. 64).

One might think that the two theses I have identified within ‘total pain’ simply map onto the two rival conceptions—the objective onto the multidimensional, the phenomenological in its thin version onto the holistic. But comparison of each pair reveals a key difference. Both the thin phenomenological conception (Mayerfeld, 1999; Edwards, 2003) and the holistic thesis (Saunders, 1964; Cassell, 1982) attend to the patient’s experience, but the former concerns how suffering feels, the latter the fact that suffering is lived as a unified experience. Likewise, both the objective conception and the multidimensional thesis identify specific causes of suffering, but they differ in what they take those causes to have in common: the objective view sees obstacles to flourishing, the multidimensional thesis sees impairments that affect the person’s overall experience. In ‘total pain’, impairments in particular dimensions matter insofar as they affect the person as a whole. The two theses are thus intelligible only in relation to one another.

## 6 Objection: how do the two theses combine?

The preceding analysis invites a final objection. If suffering results from impairments in several distinct dimensions, how can it also be experienced as affecting the person as a single whole? The worry is not that the two theses contradict each other—a scent arises from many notes, a chord from many tones, without contradiction. It is that ‘total pain’ does not, on its own, explain the passage from the many to the one. The question remains, what integrates distributed impairments into a unified experience of suffering? The present section argues that this passage is a tractable problem rather than a sign of incoherence.

The metaphor of a perfume helps frame what is needed. A fragrance is composed of distinct notes, yet what one smells is not a list of ingredients but a single scent; no individual note captures it. Suffering is likewise not a sum of impairments but a unified experience to which each contributes. Yet the metaphor also exposes a gap because a perfume does not compose itself. What, in human experience, plays the role of the perfumer—the principle that binds distinct impairments into one experience of suffering? Saunders’s writings, clinical rather than theoretical, do not say. They register that dying patients suffer as whole persons without explaining how the whole is formed from the parts.

The closest answer lies in Cassell’s account (1982). Suffering, for Cassell, occurs when a person perceives a threat to their integrity. That perception is the perfumer: it is what transforms impairments arising in different dimensions into a single experience, because each is registered not as an isolated problem but as a threat to the person as a whole. Read this way, Cassell’s theory is not a rival to the ‘total pain’ account but the first systematic attempt to supply the integrating principle that Saunders’s concept presupposes. Krikorian and Limonero (2012) extend this proposal. Suffering, on their account, arises when a person is overwhelmed—when the threats bearing on their physical, psychological, spiritual, and social dimensions outstrip the resources available to cope with them. By adding the notions of coping resources and their exhaustion, they specify a mechanism through which multidimensional impairments issue in a single, holistic experience: it is the failure of integration under load, not the impairments alone, that constitutes suffering.

Recent work in the philosophy of mind offers models of how impairments across distinct domains can integrate into a global experience of suffering. Table 1 summarizes their key features.

**Table 1:** Models of suffering and the integration of multidimensional impairments.

What is suffering?	Theoretical framework	Link between multidimensionality and holism	Reference
Perception of threat to the person	Concept of the person as a complex, multidimensional entity	The patient perceives a threat to their entire being based on how impairments across different dimensions are personally interpreted.	Cassell (1982, 1991a)
Unpleasant experience plus unmet desire	Representational theory of the mind	Unpleasant mental states arise from various physical or mental impairments. Suffering consists of an unpleasant experience together with an unmet desire for that experience to end.	Brady (2018)
Negative evaluation of the world	Philosophy of emotions	Suffering is a pervasive negative construal of one’s whole situation as calling for a change one is powerless to bring about.	Kauppinen (2019)
Disruption in mental life	Cognitive sciences, mental attention	Suffering occurs due to the disruption in mental life caused by pressing mental urgencies that relate to different aspects of well-being.	McClelland (2019)
Alienation from body, world, and values	Rich phenomenological account on suffering	Suffering is the experience of feeling alienated from the body, social relationships, or personal values.	Svenaeus (2014)

These models come from different perspectives—theories of emotion (Kauppinen, 2019), disruption models of the suffering (McClelland, 2019), the desire view of suffering (Brady, 2018), and the phenomenology of illness (Svenaeus, 2014)—and they differ in what they take suffering fundamentally to be. Yet for the present purpose their differences matter less than the questions they share. In one way or another, each locates the integrating principle (i.e., the perfumer of our earlier metaphor) in a global affection of the person’s mind or situation.

McClelland’s disruption model makes the mechanism especially explicit. Suffering, on his account, is not the unpleasant mental state itself but the unpleasant *disruption* that state produces across a person’s mental life. Pain or grief is a state that conflicts with a person’s wider mental life, and suffering consists in the disruption caused by accommodating it. When suffering captures attention, ongoing projects are impeded, other experiences are affected, and the mind must reorganize around it. An unpleasant state that causes no such disruption would not amount to suffering; pain can, in principle, be experienced without disrupting mental life. The impairment is local, but its effects are global. It is the global disruption, not the local impairment, that constitutes the suffering.

The other models trace different routes to the same destination—a perceived threat to the self (Cassell), an unmet desire for an unpleasant experience to end (Brady), and a negative transformation of one’s whole situation (Kauppinen)—but each makes distinct impairments into a single experience by having them register at a global level rather than as isolated complaints. Following the distinction drawn in Section 5.2 between the thin and rich phenomenological conceptions of suffering, Svenaeus (2014) offers a rich phenomenological conception of suffering that provides a mechanism for integration. On his model, disturbances at different levels produce a suffering mood that alters the person’s being-in-the-world, generating a sense of alienation from the familiar world and transforming their practical and evaluative possibilities. In this way, disturbances across different dimensions can coalesce into a single experience of suffering.

A detailed adjudication among these proposals lies beyond the scope of this paper, and is not necessary for the argument. What matters is that several independent research programs, from different starting points, all treat the passage from distributed impairments to a unified experience as a tractable problem, not as a contradiction to be dissolved. The dual structure of ‘total pain’ is not incoherent. Far from being a conceptual defect, its explanatory gap is a productive question at the intersection of palliative care and the philosophy of mind on suffering.

## 7 Discussion

Human suffering is the central object of palliative care, and ‘total pain’ is the concept through which the field first grasped it. I have argued that ‘total pain’ should be read as a concept with a definite dual structure: an etiological thesis of multidimensionality and an experiential thesis of holism. This structure was later systematized by Cassell’s theory of suffering as a threat to the person—often identified as the dominant theory of suffering in the field (den Hartogh, 2017; Duffee, 2020)—and elaborated in the integrated view of suffering (Krikorian and Limonero, 2012). But the architecture is Saundersian, and it remains operative in the field’s definitions, its measurement instruments, and the global health concept of serious health-related suffering.

The ‘total pain’ concept is widely reflected in the field, yet the field has not exhausted its implications. A fully holistic account of suffering points beyond the individual patient. From such a perspective, for example, family members and informal caregivers are not merely part of the patient’s environment but persons who suffer in their own right and whose interests therefore warrant separate ethical consideration (Rechenmacher, 2026).

From the multidimensional thesis, suffering can be caused by factors across various dimensions of the person; from the holistic thesis, these factors are experienced as affecting the person as a whole. Holding both theses together is what lets the field attend to the identification and treatment of symptoms while remaining centered on the person—a dual capacity that neither the objective conception, which secures intervention at the cost of autonomy, nor the thin phenomenological conception, which centers experience without securing its link to the clinical, can match. Nor is the combination incoherent: the apparent tension between distributed causes and unified experience is not a conceptual defect but an explanatory question, one that recent models in the philosophy of mind show to be tractable. This allows ‘total pain’ to inform care that is at once medically effective and ethically sound. More than 60 years after Saunders introduced it, the concept of ‘total pain’ continues to find expression across the field of palliative care.

## Footnotes

1 On Cassell’s account, what can be threatened in a person spans far more than the body. It includes who they are—their character, their history, and the meanings they attach to their illness; their web of relationships—family bonds, cultural belonging, and the social, communal, and familial roles they inhabit, since a person does not exist in isolation; their inner life—emotions, unconscious motives, habitual patterns of behavior, and a private self hidden from others; and their orientation beyond the present—their standing as a political and acting being, their projection toward the future, and a dimension many would call transcendent. Suffering, for Cassell, can arise from a threat to any of these (Cassell, 2009).

2 Physical symptoms: mild, moderate, or severe pain; weakness; fatigue; shortness of breath (dyspnea); nausea and vomiting; constipation; diarrhea; dry mouth; itching; wounds; and bleeding. Psychological symptoms: anxiety and worry; depressed mood; delirium or confusion; dementia (disorientation, agitation, or memory loss).

3 I thank Michael Rachenmacher for suggesting the distinction between these claims.
